# EHMTI-0223. Botox in the prevention of chronic migraine; 18-months follow up outcome in 67 patients

**DOI:** 10.1186/1129-2377-15-S1-G1

**Published:** 2014-09-18

**Authors:** M Khalil, H Zafar, F Ahmed

**Affiliations:** 1 Neurology, Hull Royal Infirmary, Hull, UK

## Background

Chronic migraine (CM) affects 2% of the population and Botox is the only licensed treatment for prevention of adult patients with CM.

In the UK, National Institute for Clinical Excellence (NICE) approved its use on the NHS in patients who failed three preventive medications.

NICE recommends continuing treatment beyond cycle 2 in those with 30% reduction in headache days (negative stopping rule) and the treatment is stopped when migraine become episodic (positive stopping rule).

However, the long-term outcome ie duration of required treatment remains uncertain.

## Objectives

To ascertain the duration of treatment with Botox for the prevention of chronic migraine in responders as per NICE criteria.

## Method

Adult patients with CM attending the Hull migraine clinic were offered Botox based on clinical needs and maintained a headache diary.

Data were extracted for headache, migraine, and headache-free days

Responder rate was assessed applying NICE criteria

## Results

Patients (N=67) who commenced treatment between July 2010 and June 2012 were followed up for at least 18 months.

30 Patients stopped treatment at Cycle 2 as per negative stopping rule by NICE

Of 37 responders, only 12 were still on treatment at 18 months. Nearly half of the patients stopped treatment by the 4th Cycle (positive stopping rule).

## Discussion

We continue to follow all patients who receive Botox for prevention of chronic migraine and aim to present 2 year follow up on a larger cohort at the EHMTIC September 2014.

